# The tendency of the schematic structure to maintain stability can be interpreted as mental inertia

**DOI:** 10.3389/fpsyg.2023.1243711

**Published:** 2023-11-01

**Authors:** Berlyne Wei

**Affiliations:** Psychology Department, School of Educational Science, Hunan Normal University, Changsha, China

**Keywords:** schema, unified theory framework, force, mental inertia, free energy principle

## Abstract

This paper incorporates schematic concepts related to mental inertia and provides an avenue for interpreting psychology using the principles of classical mechanics. Schemas find wide application in diverse fields, ranging from ergonomics to psychotherapy. Nonetheless, it is crucial to incorporate schemas themselves into a more unified and comprehensive theoretical framework. Drawing upon the free energy principle (FEP) and the second law of thermodynamics, it is evident that humans possess a natural inclination to construct and maintain consistent cognitive structures. This characteristic contributes to the stability of schemas within a defined range. The particular scope of the model is closely intertwined with its structure, leading to variations among individuals in diverse environments. The coherence of the schema within a defined range can be perceived as the magnitude of mental inertia. This psychological analogy emphasizes the importance of considering the influences exerted by the external environment and their effects on mental inertia when predicting the human mind and behavior.

## 1. Introduction

The overarching aim of psychological research is to predict and control human behavior. Several studies have been conducted to predict and understand human behavior, with the Theory of Mind (TOM) being one such example. The TOM posits that people’s behaviors can be predicted and understood by considering their mental states, encompassing beliefs, desires, and emotions (Wellman, 2014). However, predicting human psychology and behavior becomes intricate when attempting to account for the interplay between the environment and human adaptation within the context of understanding human cognition. Nevertheless, by likening schemas, which are structured cognitive frameworks, novel perspectives can be obtained for predicting human psychology and behavior. Based on the principles of the free energy principle (FEP) (Friston, 2013) and the second law of thermodynamics (SLT) (Carnot, 1824), schemas are systematically constructed to maintain their viability. This exhibits certain resemblances to the manifestations of inertia. The Free Energy Principle emphasizes that systems undergo changes aimed at reducing their free energy (Friston et al., 2006). Additionally, the Second Law of Thermodynamics states that energy tends to become disordered in a closed system, as it naturally seeks to disperse. Based on these two principles, when considering the individual and their environment as a whole, the internal state of the individual tends to become disordered. Consequently, the individual schema plays a crucial role as a necessary mental structure for maintaining the stability of their mental state. This implies that our focus should extend beyond understanding and explaining the mental processes and behaviors of individuals, and place greater importance on comprehending how forces and mental inertia influence changes in individuals and their recognition of existing patterns.

Schemas have been extensively applied in diverse fields, including psychotherapy and ergonomics, providing evidence of their value. The interdisciplinary significance of the unified concept of schema has been demonstrated in various domains, including interdisciplinary research on spatial schemas (Farzanfar et al., 2022), the theory does not adequately address the central core of the schematic structure, which is crucial for establishing a unified framework that encompasses multiple disciplines. The emphasis of this unified framework lies in defining a common core concept that transcends different fields of study. Through the classification of cognitive processing, the concept of schema applied in ergonomics and psychotherapy can be harmonized within a schema framework.

This paper primarily focuses on the correlation between the Free Energy Principle (FEP) and the Second Law of Thermodynamics (SLT) about schemas, as well as the application of Newton’s first law in psychology. Firstly, it will discuss the successful interdisciplinary applications of schemas in the fields of ergonomics and psychotherapy, where the term “schema” is utilized and conceptually understood in both disciplines regarding cognitive perception. Furthermore, this paper will highlight the construction of schematic structures and the predictive role of mental inertia and forces in human behavior. It also proposes that the stability of the schematic structure can be attributed to the FEP and SLT. Based on these findings, when both the thermodynamic theorem and the Free Energy Principle suggest that the schematic structure remains constant, the schematic structure can be conceptually aligned with mental inertia. This implies an advancement in the application of Newton’s laws to psychology.

## 2. The conception of schema applied in schema therapy

Schemas are organized units that contain factors including cognitive, emotional, and behavioral states, and individuals consistently exhibit the same cognitive, emotional, and behavioral patterns repeatedly (Lazarus et al., 2020). Some psychologists oppose the simplified model of conscious experience, more because the simplified model cannot account for the complexity of the visitor’s experience (Connolly and van Deventer, 2017). That complexity derives from the variability of individuals themselves and their environments, but the way individuals construct schemas is relatively simple. Schemas are formed from the development of past understandable cognitive and affective models and construct current experiences (Stein and Young, 1997). Young argues that early adverse childhood experiences are central to personality disorders, mild personality trait problems, and chronic Axis I disorders, and defines some schemas as EMS (Early maladaptive schema) (Young, 1999). Schema therapy was originally introduced into psychotherapy to complement cognitive behavioral therapy in patients with chronic personality problems due to unique personality traits that are not directly related to an Axis I diagnosis and also make them maladaptive to standard cognitive therapy (Young et al., 1996). Schema models are widely used in schema therapy (Bernstein et al., 2021), long-term psychotherapy was found to be effective in the treatment of violent offenders with personality disorders for rehabilitation.

EMSs are a persistent and widespread cognitive structure that develops based on an individual and environmental basis influenced by early life experiences and hinders subsequent successful adaptation to life (Yakın et al., 2020). Young (1990) suggests that it serves as a trait-like, stable cognitive structure that, when combined with coping responses and or health functions, results in a temporary state of emotional-cognitive behavior known as a schema model. People experience different schemas and have different schema transitions, but groups of schemas can act as a cohesive and unique unit “I” across various events and incidents (Lazarus et al., 2020), and this unique unit will further become a personal schema on the basement of metacognition.

In Schema Therapy, Schema Modes are divided into four categories, the Child Schema, the Parenting Schema, the Maladaptive Coping Schema, and the Healthy Adult Schema (Young et al., 2003) where the Healthy Adult is considered to be the mature, empathetic, and psychologically competent person who believes, senses and behaves in the schema (Edwards, 2022), the concept of maturity and “fully functioning person,” Maslow’s (Maslow, 1971) “self-actualization” and “self-transcendence,” and the traditional concept of “wisdom “(Baltes and Staudinger, 2000), among other concepts, are consistent.

By utilizing a predictive model that considers these concepts and examples, it becomes clear that it is not possible to establish a predetermined definition of behaviors and cognitions that constitute a model of healthy adulthood. Interestingly, even in some challenging environments, there is the potential for cognitive benefits to emerge. Therefore, we can only affirm that healthy adults (Edwards, 2022) possess the ability to engage in “responsible, emotionally connected, and tolerant communication,” while also demonstrating “reciprocally consistent communication,” and exhibiting a “democratic, consistent, and coherent identity.” This presents a dilemma in which we can only speculate about the underlying mechanism based on the observed symptoms, rather than accurately predicting the symptoms based on the mechanism. Viewing it from a schematic perspective, a psychologically healthy adult is sufficiently mature to understand their schema and can adjust it to suit the environment. Therefore, it can be argued that when an individual’s schema fails to align with the environment, there is a significant probability that they will experience adverse psychological symptoms in the future.

Based on the theoretical framework of forensic Schema Therapy (ST), criminal and violent behaviors can be comprehended by examining schema models or sequences of momentary states that encompass emotions, cognitions, and behaviors. These schema models can predict future violations of behavioral regulations (Keulen-de Vos et al., 2016). These research studies highlight the predictive role of schemas. Nevertheless, the current research on the mechanisms underlying changes in psychiatric symptoms faces a limitation in that the hypotheses have yet to be experimentally validated (Hoyer et al., 2017). At present, the hypothesized mechanisms are predominantly evaluated using trait instruments rather than State Aggression measures. This shift in approach places greater emphasis on predicting symptoms based on the present state, rather than predicting the mechanisms that give rise to these symptoms (Yakın et al., 2020). This statement suggests the importance of investigating a mechanism that can effectively predict the onset of a disease. Furthermore, it emphasizes the need for a unified framework that focuses on understanding the mechanisms involved in predicting symptoms.

## 3. The conception of schema applied in ergonomics and automatic processes in cognition

Schema theory demonstrated to be successfully applied to core domains of ergonomics, including SA (Situation Awareness), NDA (Naturalistic decision-making), and error research. It is believed to be potentially the theoretical foundation for the unification of ergonomics (Plant and Stanton, 2013). Schema theory is also broadly applied in ergonomics, including human–computer interaction (Chalmers, 2003), tool use (Baber, 2006), military applications (Stanton et al., 2006; Stewart et al., 2008), and a variety of transport domains including road (Walker et al., 2011), rail (Stanton and Walker, 2011), and aviation (Plant and Stanton, 2012). However, Schema theory has received criticism for the lack of consensus on its definitions and the absence of a unified theory (Plant and Stanton, 2013). One of the major criticisms directed toward schema theory is the lack of a unified theoretical framework that encompasses all dimensions of the theory (Lodge et al., 1991). A theory must be capable of both predicting and explaining world phenomena (Kerlinger and Lee, 2000). For instance, a study revealed that individuals who hold both motorcycle and car licenses have a decreased probability of being involved in car-motorcycle accidents in comparison to those who possess only a car license (Magazzù et al., 2006). According to schema theory, individuals with a car-only license exhibit greater cognitive incompatibility between car drivers and motorcyclists due to the absence of the necessary schema to effectively manage diverse road scenarios (Walker et al., 2011). This example is also seen as a predictive role of schema theory (Plant and Stanton, 2013). Schema theory provides insights into the function of schema organization in the mind, acting as mental templates that guide attention towards perceived information. The active and adaptable nature of schematic structures allows incoming information to shape and modify schemas, ultimately influencing perception (Neisser, 1976). In conceptual terms, Neisser defines schemas as organized mental structures of thought and behavior that are utilized to organize knowledge of the world (Neisser, 1976). Meanwhile, schemas are structured units that encompass cognitive, emotional, and behavioral states. Within these schematic structures, individuals consistently and repeatedly demonstrate the same cognitive, emotional, and behavioral patterns (Lazarus et al., 2020). Although the two conceptions of schemas stem from different domains, they demonstrate striking similarities, except for variations in emotional aspects. This conceptual alignment serves as the basis for establishing a unified construct.

The hierarchical nature of schemata plays a crucial role in their functioning. One of the fundamental assumptions is that actions can be specified at the highest-level schema, and once this activation occurs, lower-level schemata autonomously complete the action sequence (Norman, 1981). It is worth emphasizing that the term “nodes” should not be interpreted as physical structures. Instead, it refers to mental representations and their interconnections. The use of nodes and associations serves as a metaphorical representation of the structure and linkage of knowledge within the mind (Plant and Stanton, 2013). Furthermore, Minsky’s Frame Theory (Minsky, 1975) posits that human intelligence is derived from the top-down processing of stored background information, serving as a complement to organizational structures. Similarly, Schank and Abelson’s Script Theory (Schank and Abelson, 1977) views schemas as the embodiment of procedural knowledge, offering an explanatory perspective for schema theory and its practical applications. Different from the script (Albarracin et al., 2021) that it takes a different approach by considering scripts as sequences of events in a given type of situation, without employing the active inference apparatus to construct a formalization of scripts.

From a conceptual perspective, the application of schema in ergonomics exhibits a remarkable similarity to automatic processing in cognitive processes. According to Shiffrin and Schneider, cognitive processes encompass both voluntary and automatic processes, with the latter not being governed by arbitrary control, not emphasizing the limitations of the system’s capacity, and not necessarily demanding attention (Shiffrin and Schneider, 1977). Anderson highlighted that a variety of automatic processes can be conditioned to respond to stimuli and prioritize historical task relevance. These processes exhibit quick and flexible adaptations to facilitate goal-directed behavior. Additionally, Anderson suggested that the conceptualization of automatic processes may extend beyond our conventional understanding of cognitive control (Anderson, 2018).

Although we categorize unconscious processes as automatic and conscious processes as voluntary, then Wiggins (2020) “it now seems equally credible that goals experienced as conscious decisions are generated non-consciously, and then enter conscious awareness, as the more intuitive converse.” Automatic and voluntary processes are inherently interconnected aspects of cognition, and it is conceivable that automatic processes carry greater weight in predicting the human mind and behavior. The repetitive nature of automatic processes, combined with the purposeful adaptations of schematic structures to establish equilibrium with the environment, contribute to the predictability of both repetitive behaviors and cognitive processes, especially when the environment remains relatively stable. Several studies have discovered that human decision outcomes are determined before conscious awareness, implying that automatic processes exert a more significant influence on cognition than previously believed. This plausible result aligns with existing research findings (Soon et al., 2008). Although automatic processes seem highly relevant to psychological prediction, they can sometimes be misleading. This is due to the close integration and strong correlation between automatic and voluntary processes, which are significantly influenced by individual environments. Consequently, it is erroneous to consider automatic processes as the sole focus of predicting human psychology and behavior comprehensively. The role of schematic structure becomes apparent in the predictive capacity of automatic processes when individuals operate within stable and unchanging environments, where significant changes are unnecessary. The schematic structure, resembling mental inertia, tends to maintain existing actions and, to some extent, supports the persistence of automatic processes. However, when external forces exert a substantial impact or other factors come into play, individuals may change their behavioral states or modify their behaviors by adjusting their schemas. During such instances, the predictive function of automatic processes diminishes, necessitating consideration of additional factors. Undoubtedly, the analogy between the size of the schematic structure and the magnitude of mental inertia serves as the primary step in considering multiple influences during the process of psychological prediction. This analogy helps eliminate many influences that are inadequate to bring about behavioral change. Furthermore, learning provides a pathway to explore the connection between automatic and voluntary processes. Learning is a transfer from one system to another (Sigman et al., 2005; Sigman, 2017) and the process of structuring schemas encompasses both of these systems. Mariano Sigman put forward the notion that learning encompasses the transfer of knowledge between different systems, specifically involving the shift from voluntary control (cortical dorsal system) to automatic control (ventral system) (Sigman et al., 2005; Sigman, 2017). In this scenario, the two cognitive processes are closely intertwined. In the realm of cognitive analysis, drawing an analogy between automatic processes and the concept of psychological inertia, and further, likening voluntary processes to forces symbolizing the influx of information, illuminates the perspective that the process of learning can be conceptualized as a force transcending psychological inertia. Consequently, this cognitive force propels individuals towards active engagement in learning behaviors. Such engagement yields a spectrum of potential outcomes, notably including the expansion of schematic structure. The objectives of learning can be seen as an iterative interplay between voluntary control and automatic processing. Through this iterative process, continuous learning results in an enhancement of automatic processes, which form an integral part of the schematic structure.

Nesser’s PCM perspective (Cyclical Perception Model) explains the cyclical process of interaction between individuals and the environment. In this model, the schemas held by individuals serve as predictive mechanisms in perception, exerting influence on decision-making and directly impacting actions. Hohwy mentioned PEM (prediction error minimization) (Hohwy, 2016) which means that the brain continuously minimizes the error between the predicted sensory input based on the brain’s model of the world and the actual sensory input (Friston and Stephan, 2007). We can further construe PEM as the proactive pursuit of information by the individual to refine their schematic structure, thereby molding them into patterns that align most harmoniously with the environment. This process, in turn, serves to bolster cognitive inertia or diminish the propensity for compelled individual adjustments. However, we have some studies that find that human decision-making is not entirely rational (Kahneman, 1979) although these studies are not sufficient to disprove the argument that humans make rational decisions. For humans, the rational construction of our schema represents the closest approximation to the mentioned healthy adult schema, showcasing the highest level of maturity. In this well-developed schema, both automatic and voluntary processes work harmoniously. Early Maladaptive Schemas (EMS) serve an adaptive role in situations where rationality is not fully developed or fails to function effectively. Nevertheless, it is crucial to recognize that relying solely on EMS may hinder a smooth and successful life in the long term, rather than serving as a sustainable solution. Building upon this premise, considering the intricate nature of individuals and their environment, as well as the multitude of confounding variables arising from their interaction, the schema-based prediction model highlights relatively consistent schematic structures as enduring variables. To bring about changes in a schematic structure, an equivalent or greater force is necessary, which can be achieved through modifications in the voluntary control system or alterations in the external environment. When focusing on Early Maladaptive Schemas, it is reasonable to expect that as the complexity of the encompassing schema increases, the magnitude of force required for transformation also intensifies.

Consequently, this paper aims to minimize discussions on factors such as culture and other aspects of schematic structure, focusing primarily on exploring schematic structure itself and providing a rationale for analogizing it to mental inertia. Nevertheless, the scope of this framework extends beyond being a unified theoretical foundation solely for ergonomics. It can serve as a comprehensive framework for studying the human mind and behavior across various domains. This is accomplished by drawing an analogy between the schema and the notion of mental inertia.

## 4. Schematic structure and mental inertia and force

In the context of transportation, mental inertia can be defined as the occurrence of unintentional, goal-directed repetitive behaviors, mental representations, and automatic behavioral responses (Sommer, 2011). In sociological and psychological contexts, mental inertia is recognized to possess three distinct properties: the ability to learn from repeated behaviors, being triggered by content-related or script-based processes, and ultimately giving rise to the manifestation of automatic behaviors (Haggar et al., 2019). It is assumed that mental inertia and habituation have similar meanings and are interchangeable in diverse literature (Gao et al., 2020).

If we not only conceptualize the comparison between classical physics and psychology but also take into account their practical application, it becomes crucial to establish a relationship between psychological schemas and physical mass. According to Newton’s first law (Newton, 1848), an object will maintain a state of rest or motion unless a force is exerted. The development of human mental schemas is influenced by environmental stimuli, which can be regarded as forces, while the magnitude of human mental schemas can be seen as a form of mental inertia. In 1872, Ernst Mach posited that the inertial mass of a particle is somehow influenced by the presence of other masses in the universe (Mach, 1872), an idea that influenced Volkmar Putz’s contention that the inertia of a mass is a phenomenon exclusively induced by the gravitational influence of other masses (Putz, 2019). In the context of this analogy, it becomes evident that individual schemas are influenced by environmental stimuli. Following Newton’s first law, if the external stimuli surpass the mental inertia, individuals will undergo corresponding changes in their mental structures, ultimately leading to behavioral modifications. Conversely, if the stimuli are insufficient to surpass the mental inertia, no significant changes in mental or behavioral patterns will occur. However, it is vital to avoid linking mental inertia exclusively to specific behaviors. Rather, it reflects an individual’s tendency to maintain recurring behaviors, which may be associated with specific behaviors but should not be narrowly confined to them. If schema models are considered trait-type stable cognitive structures (Young, 1990), then they can be measurable.

In the field of psychology, the PMT (Psychological Momentum Theory) hypothesis suggests that changes in response rate are directly linked to the magnitude of the intervention or stimulus. It is worth noting that this hypothesis does not provide a clear analogy to the concept of momentum (Deemer et al., 2021), but rather uses the response rate (velocity) to define mass (inertia). According to their viewpoint, individuals who do not actively pursue a specific task are characterized as being in a low-momentum state or having a low degree of momentum. Conversely, individuals who make efforts to pursue a given task are defined as having a high degree of momentum or being in a high-momentum state. A problem arises when trying to measure an individual’s mental inertia solely based on behavioral response rates, as this does not provide a comprehensive definition of mental inertia. From the individual perspective, the given task is seen as a force that is used to alter the motor state, and regardless of whether the state one maintains is related to the given task, we cannot determine whether the high momentum state (the state of pursuing the given mission) that the individual maintains after the altered motor state originates from his/her high mental mass or high velocity. This is the most important point because momentum is equal to mass times velocity (Markman and Guenther, 2007). As a result, these two factors exert an equal influence on psychological momentum. It is argued that if an individual manages to reach a state in which he/she performs an activity at a high frequency, it will be easier to continue performing that particular action (i.e., high inertia or large HMSI) (Deemer et al., 2021). This view of the state is plausible, but there are some problems with the definition of mental inertia. The correct characterization of this state implies that if an individual manages to consistently engage in an activity with a high frequency, they should be deemed to possess a state of high velocity. Additionally, the longer they can maintain this state without being influenced by external forces, the greater their mental inertia, which can be described as a high level of High Mental Inertia (HMSI). It is crucial to recognize that if an individual effortlessly enters a state of performing an activity with a high frequency, their mental inertia would be low.

## 5. Expansion of schematic structure and increase in mental inertia

Conditioned reflexes can contribute to the process of schema construction. The establishment of conditioned reflexes is not restricted to specific observable behaviors but also influences the development of schemas. Mental inertia is represented by the degree of value, importance, and timeliness that an individual places on a particular behavior with situational variables (Markman and Guenther, 2007). An escalation in the perceived importance of a certain object or behavior contributes to an augmentation of psychological inertia. Here is a corollary regarding mental inertia and schematic structure. Taking Pavlov’s dog as an example, before the establishment of the conditioned reflex, the dog did not respond to the bell, indicating that the ringing held no significance for the dog. However, after completing the conditioned reflex training, the dog’s response to the bell became important. Consequently, the schematic structure of bell food salivation appeared within the dog’s cognitive framework. The ringing transitioned from being inconsequential to significant. According to the definition of mental inertia mentioned earlier, mental inertia expands. The role of Pavlov’s conditioned reflex is not solely attributed to the association formed between the conditioned and unconditioned stimuli due to their proximity. It is more importantly driven by the conditioned stimulus reliably anticipating the appearance of the unconditioned stimulus, which is regarded as the learning of relationships between these events (Rescorla, 1988). This learning of relationships between events shares similarities with the formation of schematic structures. However, it is crucial to acknowledge that conditioning represents just one among numerous learning processes (Rescorla, 1988). Furthermore, we can observe that the pursuit of meaning behind the bell sound is futile since it ultimately points to food, which serves as the fundamental source of meaning for the bell sound. Therefore, if we aim to control the dog’s consistent response to the bell sound, it is enough to ensure that the association between the bell sound and food remains intact within the dog’s environment, without requiring attention to the dog’s “mental process” or other factors. The establishment of the association between bell sounds and food not only enriches the schematic structure of dogs but can also be interpreted as an augmentation of mental inertia. As a result, animals develop stronger resistance to analogous connections between conditioned stimuli and unconditioned reflexes. This inference can also explain why the rats (Kamin, 1969) failed to acquire anticipatory responses to light, indicating that the effects generated by similar stimuli (light and sound) are no longer capable of modifying the rats’ already elevated mental mass. In other words, the heightened mental mass strengthens the rats’ resistance to other stimuli. When a human schema is formed without additional information input or voluntary processes, the schema tends to remain relatively stable. Due to the presence of mental inertia, it spontaneously resists the influence of information below its threshold. This resistance is manifested in the persistence of current behaviors. This suggests that if an external force (information) is not sufficiently strong, it will not induce a modification in the schema, resulting in no mental or behavioral changes. Conversely, when an external force does bring about a mental or behavioral change, the schema will either remain unaltered or undergo an increase, leading to an escalation or preservation of mental inertia.

According to Piaget, in the process of cognitive development, the construction of schemas or structures unfolds by progressing from simple to complex forms. Furthermore, Piaget highlights that the acquired structures are subservient to the ongoing development of the structures themselves (Piaget, 1964). It is justifiable to assert that the conditioned reflexes present at birth form the basis for the establishment of schematic structures and the presence of mental inertia. On one hand, this established relationship constantly receives input from the external world and actively constructs its schema. The initial conditioned reflex of an infant is a tendency to respond for survival. One of the remarkable strengths of a newborn is the complete set of useful reflexes, with certain conditioned behavioral patterns referred to as survival reflexes due to their significance in ensuring survival (Berne, 2003). Moreover, some conditioned reflexes also establish a positive bond with the caregiver, such as sucking on the nipple (Shaffer and Kipp, 2013), on the other hand, once a schema is formed and solidified, it tends to resist any forces that may disrupt it. The infant or the conditioned reflex is not concerned with who establishes this inclination but rather whether it can be established. And once the schema is reinforced through repetitive experiences, it becomes stable in its response.

## 6. An alternative explanation for the tendency of the structure shown to maintain stability

The tendency of individuals to preserve the stability of the depicted structure is influenced by the interplay between the free energy principle (FEP) and the second law of thermodynamics. The second law of thermodynamics postulates that the energy of an isolated system has a tendency to be disordered, and the concept of entropy is used to describe the degree of disorder of this energy, which also corresponds to the degree of organization of a particular given system (Rabeyron, 2021). Biological systems are thermodynamically open because they exchange energy and entropy with their environment (Friston et al., 2006). Therefore, living organisms endeavor to sustain a reduced level of entropy by actively engaging with the environment. From this perspective, living systems mitigate internal entropy by “externalizing” it to the surrounding environment, thereby expelling disorder and upholding internal order (Rabeyron, 2021). In neuroscience, this principle is known as the “free energy principle” (Rabeyron, 2021). In 1969, Stoker introduced a hierarchical model that encompasses three levels: the inorganic domain at the lowest level, the organic domain at the intermediate level, and the information domain at the highest level (Stoker, 1969). Building upon this notion, Grobbelaar posited that these hierarchical relationships play a crucial role in establishing the individual as a cohesive entity, with each lower level of organization defining the boundaries and parameters for the subsequent level (Grobbelaar, 2012). In essence, this can be perceived as a constraint that influences the higher levels based on the lower levels. Connolly and van Deventer have put forth a three-level recursive description of psychoanalytic conditioning principles (Connolly and van Deventer, 2017) influenced by Grobbelaar’s description (Grobbelaar, 2012) (and a similar model found in Connolly (2016)), which contains the inorganic (atoms and molecules), organic (cells, tissues, organs, systems, and organisms), and psychological layers (self-defense and pleasure principles).

In 2013, Friston proposed the notion of the “Markov blanket,” enabling the exchange of the system’s internal state with its external state (Friston, 2013). This “Markov blanket” is essential for effectively separating the internal and external states, necessitating the preservation of minimum free energy within the internal state. Furthermore, Friston introduced the concept of a “working model” or “generative model” that allows individuals to simulate the environment through predictive mechanisms. By enhancing expected outcomes, this approach fosters organismal development and leads to a reduction in internal entropy (Friston, 2009). At each layer of the system, the formation of the internal state triggers the role of the Markov blanket as a boundary, effectively distinguishing the internal and external systems. For example, as the organic system emerges within the inorganic system, the Markov blanket acts as a boundary, separating the organic and inorganic components. Similarly, at the mental level, the Markov blanket serves to segregate internal mental processes from external chaotic stimuli, establishing an organized mental structure. Since each domain operates under the free energy principle, which drives an inherent tendency for increasing entropy and returning to a lower level, the preservation of the Markov blanket’s state becomes crucial. While the free energy of the working model may not be minimized, its viability rests on being below the external free energy. This requirement entails the establishment of an organized and stable structure, with the working model’s inherent tendency to maintain internal stability serving as the cornerstone for upholding stability within the personal schema. Kuperberg proposed a hierarchical framework that contributes to our understanding of cognitive mechanisms, emphasizing their strong correlation with the generation and acquisition of sequential movements (Kuperberg, 2021). In the case of schemas, these investigations primarily concentrate on the process and content of construction, rather than the resulting implications. In my view, the free energy principle pertains to the idea that the primary objective of schema construction is to minimize internal entropy as much as possible.

Freud’s death drive is considered a tendency for living matter to gravitate toward inorganic matter (Armengou, 2009), which means that if the death drive acts on the Markov blanket, it will continue to be acted upon by the death drive, and if the Markov blanket loses its function, the individual will not be able to stabilize his or her psychological state. Rabeyron proposed that just as cellular structures need closed boundaries to ensure their survival, mentally such closed structures can distinguish between the inside and the outside (Rabeyron, 2021), but such closed structures also need to maintain a certain exchange with the environment and keep their structures stable during the exchange, and we can consider this as the reason for schema construction. It has been suggested (Tran The et al., 2020) that if the entropy of the universe tends to increase to a maximum, according to Clausius’ theory, the closer the universe gets to the limit state, the more the possibility of new changes will disappear and when this state is reached, there will be no further changes and the universe will find itself in a “state of continuous death” (Clausius, 1868; Locqueneux, 2009). These considerations make it possible to reread the hypothesis that Freud had about the death drive, “If we apply to live organisms this tendency of the universe toward a state of equilibrium, defined by the irreversible absence of all discernible motion and all difference in tendency – in other words, equivalent to a definitive death – we can envisage a closed system consisting of the unit “organism-environment.” The “organism-environment” can also be considered psychologically as the life space (Levin, 1936) of Levin, and these ideas are in agreement. The definition of life and death (Tran The et al., 2020) “according to Prochiantz, also be interpreted as an argument against any concept of life as a singular point of resistance to the second law of thermodynamics. That is to say, as a structure that, at a given point, opposes increasing entropy (Prochiantz, 1990).”

Levin asserted that psychological events are intricately influenced by the simultaneous state of both the individual and the environment (Levin, 1936). Unlike physics, which deals with singular closed systems and applies Newton’s laws of motion, psychology examines multiple open systems (Heidbreder, 1937). The schematic structure, like the mental topology, operates within defined boundaries and exhibits a semi-closed nature. However, it is crucial to distinguish that the schematic structure is not an exact representation of the entire topology. Rather, it encompasses the repeated and organized elements within the mental topology. By mitigating the propensity for entropy to escalate within the internal system, the schematic structure serves as a stabilizing force. Rabeyron (2021) cited “the aim of all life is death and, looking backward, inanimate things existed before living ones” (Freud, 1920). From this perspective, it is posited that death, representing an inorganic state, is an intrinsic and indispensable aspect of life, which is characterized by its organic nature. Moreover, it is suggested that every living entity can be perceived as a mortal being, existing and evolving within the dimension of time. The previously mentioned “Markov blanket” serves a dual role. On one hand, it functions as a connecting link between the internal and external aspects of the system. On the other hand, it serves the purpose of delineating and distinguishing the internal structure from the external environment (Friston, 2013). Mental structures are restricted by the organism (Connolly and van Deventer, 2017). These constraints not only limit the formation of mental structures by exerting a force that dissolves them, but they also generate a counteracting force that organizes their structure, thereby maintaining the overall integrity of the mental structure. Bernfeld and Feitelberg attempted to elucidate mental functioning through the lens of entropy, providing experimental evidence that the determinants of human behavior are partially governed by the principle of entropy (Bernfeld and Feitelberg, 1931). Rabeyron argued that entropy gives rise to a “momentum or impulse” in inanimate matter, leading to the emergence of organizational structures through successive transformations. From this perspective, entropy does not directly impact the organism or the mental state but instead serves as a regulatory mechanism for the living being, as Thomas Rabeyron quotes Freud (1924) for the death drive turning outward to produce “destructive instinct, the instinct for mastery, or will for power for mastery, or will for power.” The concept of the death drive, in itself, does not carry any intrinsic value or additional connotations. From the perspective of conditioned reflexes, it simply serves as an unconditioned stimulus to which individuals respond instinctively. When the unconditioned reflex associated with death becomes linked with other stimuli or behaviors that lead to death, individuals will naturally seek to avoid such stimuli or behaviors. The organism exhibits a strong reaction towards death, which, in my view, can be considered an unconditioned response. From an entropy perspective, an organism can be deemed “dead” when it no longer maintains a decrease in internal entropy or when the Markov blanket loses its effectiveness, allowing for unrestricted exchange of various substances and energy with the environment.

Taking an alternative perspective on Freud’s statement regarding death as a state of life, we can posit that living beings are not driven solely by the pursuit of entropy reduction. Instead, they aim to mitigate or temporarily halt the increase in entropy. Furthermore, living beings can be perceived as a continuous process of transformation instigated by entropy – a phenomenon that extends beyond organic matter to encompass inorganic structures (Rabeyron, 2021). The mental hierarchy, constrained by the living organism, serves the purpose of not only assisting the organism in its pursuit of slowing down or temporarily suspending entropy but also exhibiting its inclination to decelerate or suspend. This tendency leads the mental hierarchy to make decisions that aim to mitigate or pause the incessant increase in free energy, rather than actively seeking a decrease in free energy. Consequently, this can be likened to the deterministic inclination or deterministic impact that individuals experience when making decisions (Kahneman, 1979).

## 7. The unified framework should be studied beyond the mental processes

Based on this rationale, when attempting to predict the human mind and behavior, it is advisable to shift the focus away from cultural discussions, as mentioned earlier. Instead, emphasis should be placed on understanding the individual’s schematic structure and mental inertia, rather than delving extensively into the specifics of their schematic content. While exploring the schematic structure and mental inertia can provide valuable insights for analysis, their direct applicability in accurately predicting human behavior is limited. Although quantifying the magnitude of mental inertia may pose challenges, and expressing the magnitude of the schematic structure in simple weights may not be feasible, we can define the boundaries of the schematic structure with the “Markov blanket.” The Markov blanket represents a relatively stable structure established by an individual within their life space. Life space is the sum of facts that determine a person’s behavior at a given moment (Levin, 1936). The life space referred to here encompasses the schematic structure, which represents the self-perceived structure within an individual’s life space. The close connection between the schematic structure and mental quality enables the quantification of the size of the schematic structure through the concept of mental inertia.

Some psychologists argue against the simplistic model of conscious experience, primarily due to its failure to capture the intricate nature of the force-visitor experience (Connolly and van Deventer, 2017). By considering schema stabilization and schema magnitude, attention can be directed towards assessing how an individual can alter their current circumstances or predicting their capacity to navigate challenges, without delving extensively into the complexity of their subjective experience. While analyzing past experiences of individuals is valuable for constructing schemas, the ultimate aim is to understand how the human mind and behavior can be transformed and how behavior and the psychological process can be effectively controlled (Gerrig and Zimbardo, 2002).

This suggests that research on consciousness and behavior is not inherently meaningless. Instead, it suggests that we can surpass the description and explanation of mental processes and approach human psychology and behavior from a predictive and control standpoint. Thagard contends that the holistic understanding of human cognition can be seen as a complex mathematical model, necessitating the use of differential equations for its construction. Furthermore, human cognition can be viewed as a chaotic model, wherein even a seemingly insignificant variable can result in substantial alterations (Thagard, 2005). However, this model of chaos can somewhat align with our expectations if we limit its application to human schemas within defined topological boundaries. We must shift our attention away from the subtle changes occurring within the model and instead concentrate on how schemas maintain their structure and undergo regular transformations within this intricately operating life space. By directing our attention to the consistency of the schematic structure in specific conditions and the forces that impact it, we can make accurate forecasts regarding changes in human behavior and psychology. It is essential to emphasize that forces do not inherently modify human psychology and behavior; rather, the observable changes in behavior and psychology are a result of these forces. Furthermore, it is important to recognize that these forces predominantly stem from the external environment’s influence, while internal forces facilitate the development of a healthy adult schema within an individual.

## 8. Modifying early maladaptive schema

Maladaptive schemas arise from unmet core emotional requirements in early childhood and recur throughout our lives (Young et al., 2003). There is an argument suggesting that although some aggressive and risk-taking behaviors in childhood may have negative psychological implications, from an evolutionary perspective, children raised in unpredictable and challenging environments rely on such behaviors for their survival (Ellis et al., 2009, 2017). For children to thrive in a specific environment, prioritizing short-term effective strategies over long-term well-developed ones is a more adaptive choice (Bjorklund, 2018). The production of maladaptive schemas can, in certain respects, provide adaptive benefits. In harsh environments, it is probable that individual schema, under the influence of PEM, will undergo transformation into EMS.

A harsh and unpredictable environment does not have a universal detrimental effect on intellectual functioning during child-rearing. Instead, it can lead to diverse outcomes for different aspects of executive functioning (Mittal et al., 2015). According to the mental health model perspective, the growth process can lead to negative outcomes, including insecure attachment, sexual promiscuity, and academic and economic failure. However, this perspective primarily emphasizes the costs to children while overlooking their potential adaptive benefits (Bjorklund, 2018). Conversely, an evolutionary developmental perspective, grounded in life history theory, emphasizes that early development is relatively less disrupted by stressful environments and instead guides or regulates the establishment of adaptive strategies that can effectively function in such harsh conditions (Ellis et al., 2017). This implies that the distinction between maladaptive schemas and healthy adult schema models goes beyond their potential association with later psychological disorders. More crucially, it lies in their capacity to facilitate long-term adaptation to varying circumstances. Consequently, the content of individuals’ constructs may differ depending on their environment and individual differences, which can contribute to the development of psychological disorders. Best-fit environmental conditions play a crucial role in facilitating enhanced cognitive development in individuals. Both deaf and non-deaf children of the same age living in environments that matched their own were able to pass the theory of mind task, whereas deaf children with hearing parents showed significant delays in the theory of mind development (Peterson, 2009). Gaining an understanding of schema formation and recognizing the adaptive role they play can significantly enhance our comprehension of individuals. Assisting individuals in transitioning from maladaptive schemas to healthy adult schemas necessitates a focus on their specific schemas and identifying the forces required for transformation. Addressing the harmful effects of poor schemas on psychological well-being may necessitate distinct forces to induce change, as simply understanding the content of the schematic structure does not bring about automatic modification. The complexity of the graphical structure corresponds to the level of force required to instigate change. If there are insufficient forces capable of driving substantial schema transformation, the individual’s difficulties will persist, and even modifying cognition and behavior alone will not lead to a successful resolution of their problems.

## 9. Discussion

The purpose of this paper is not merely to superficially link the disciplines of psychology and physics through the concept of schema. Instead, it aims to apply Newton’s laws, which have been extensively employed in physics, to reevaluate psychological research from a different perspective within the field of psychology. Typically, we tend to understand individuals and their mental processes from the TOM perspective. However, due to factors such as the environment and individual differences, we sometimes cannot fully comprehend an individual’s beliefs, desires, and emotions. This limitation can pose challenges in the application of psychology. By shifting our perspective to not only understand an individual’s beliefs, desires, and emotions but also to comprehend individual mental processes and behavior through the lens of classical mechanics, specifically through the relationship between inertia and schematic structures, or by employing different forces to assist individuals in modifying EMS, it may be possible to better facilitate individuals’ psychological well-being.

In predicting human behavior, a sole TOM-based understanding of individuals may not suffice, as humans continuously interact with their environment. From the perspective of forces and inertia, particularly if Newton’s laws can be integrated to aid in predicting human behavior, much like predicting the trajectory of an object’s motion, this approach can significantly benefit psychological research.

One aspect that this paper aims to explore is the relationship between the expansion of schematic structures and the increase in psychological inertia. It is essential to consider how to measure the magnitude of psychological inertia associated with corresponding schematic structures and how to apply mental inertia to Newton’s laws of motion. These considerations should be addressed in future research efforts.

In conclusion, schematic structures hold significant potential for providing a unified framework in psychology. If a psychological theory can offer value across diverse cultures, encompassing both collectivist and individualist cultures, as well as various academic disciplines, it represents a step towards achieving a unified framework. Once a method for quantifying and describing schemas is established, it becomes easier to conduct interdisciplinary research and approach psychology from various perspectives. Unlike attempting to reduce complex mental processes to mechanical interactions, schema theory acknowledges the influence of contextual constraints on human behavior, cognition, and individuals themselves. While this constraint enables the prediction of certain aspects of human behavior and cognition, it does not imply precise prediction of all human minds and behaviors. A complete schematic structure, from a psychological standpoint, corresponds to a healthy adult model capable of effectively navigating various situations in their environment without additional assistance. As a practical application, schema theory should be implemented with consideration for measuring psychological attributes and assessing the magnitude of forces at the psychological level.

## Data availability statement

The original contributions presented in the study are included in the article/supplementary material, further inquiries can be directed to the corresponding author.

## Author contributions

The author confirms being the sole contributor of this work and has approved it for publication.
